# Supplementary material for: Adaptive evolution of cytochrome b in songbirds
Source: Biol Open. 2025 Apr 14;14(4):bio061908. doi: 10.1242/bio.061908 (PMC12032549; doi:10.1242/bio.061908)
Supplement: Supplementary information [file biolopen-14-061908-s1.pdf]

Dataset S1. Comparison of mitochondrial ROS between songbirds and other modern bird from the results of Delhaye et al 2016, Table1

Available for download at
https://journals.biologists.com/bio/article-lookup/doi/10.1242/bio.061908#supplementary-data

Dataset S2. Comparison of genetic distances for bc1 subunits between songbird and other modern birds

Available for download at
https://journals.biologists.com/bio/article-lookup/doi/10.1242/bio.061908#supplementary-data

Dataset S3. Songbirds-specific substitutions in cytochrome bS8N, P10Q, S18A, S26P, A30T, M54T, S61N, T70M,L,V, N73D, Y76F, Y110N, T116I,V,A191V, L210ST, F225Y, K310T, Q313L, I328A, T370A, N379K, Y380L.

Available for download at
https://journals.biologists.com/bio/article-lookup/doi/10.1242/bio.061908#supplementary-data

Dataset S4. Songbirds-specific substitution in cytochrome c1K34R, Y90H, S129A, P137A, N166D, R203K, S217P, S228A.

Available for download at
https://journals.biologists.com/bio/article-lookup/doi/10.1242/bio.061908#supplementary-data

Dataset S5. Songbirds-specific substitutions in Rieske protein M19Q, T23A, I27G, D31S, K116Q, Y178L, T184V,H.

Available for download at
https://journals.biologists.com/bio/article-lookup/doi/10.1242/bio.061908#supplementary-data

Dataset S6. Songbirds-specific substitutions in the 14KDA subunit R17C, Y38N,H, L102Q, P88R,H, K87H

Available for download at
https://journals.biologists.com/bio/article-lookup/doi/10.1242/bio.061908#supplementary-data
